# Carboxylic Terminated Thermo-Responsive Copolymer Hydrogel and Improvement in Peptide Release Profile

**DOI:** 10.3390/ma11030338

**Published:** 2018-02-26

**Authors:** Zi-Kun Rao, Rui Chen, Hong-Yu Zhu, Yang Li, Yu Liu, Jian-Yuan Hao

**Affiliations:** School of Microelectronics and Solid-State Electronics, University of Electronic Science and Technology of China, No.4, Section 2, North Jian’she Road, Chengdu 610054, China; raozikun@outlook.com (Z.-K.R.); jn717@163.com (R.C.); 18200203805@163.com (H.-Y.Z.); qianzhuodichang@outlook.com (Y.L.); poly1634@hotmail.com (Y.L.)

**Keywords:** carboxylic termination, hydrogel, peptide release

## Abstract

To improve the release profile of peptide drugs, thermos-responsive triblock copolymer poly (ε-caprolactone-co-p-dioxanone)-b-poly (ethylene glycol)-b-poly (ε-caprolactone-co-p-dioxanone) (PECP) was prepared and end capped by succinic anhydride to give its carboxylic terminated derivative. Both PCEP block copolymer and its end group modified derivative showed temperature-dependent reversible sol-gel transition in water. The carboxylic end group could significantly decrease the sol-gel transition temperature by nearly 10 °C and strengthen the gel due to enhanced intermolecular force among triblock copolymer chains. Furthermore, compared with the original PECP triblock copolymer, HOOC–PECP–COOH copolymer displayed a retarded and sustained release profile for leuprorelin acetate over one month while effectively avoiding the initial burst. The controlled release was believed to be related to the formation of conjugated copolymer-peptide pair by ionic interaction and enhanced solubility of drug molecules into the hydrophobic domains of the hydrogel. Therefore, carboxyl terminated HOOC–PECP–COOH hydrogel was a promising and well-exhibited sustained release carrier for peptide drugs with the advantage of being able to develop injectable formulation by simple mixing.

## 1. Introduction

In the last decades, protein and peptide drugs have become an important class of therapeutic agents due to low side effects, specific targeting site and low dosage. However, the available protein or peptide drugs are easily hydrolyzed by enzymes in vivo, leading to a short biological half-life period. Harsh preparation conditions, such as the aqueous/organic interface produced by a water-in-oil microemulsion may also induce protein denaturation and aggregation [1]. Although controlled drug delivery systems (CDDS) have been researched to arrive at an improved functional in vivo use drug carrier, setting up a simple and satisfying solution enabling drug loading at mild conditions remains a challenge. Excellent CDDS should be expected to afford abundant advantages like improved efficacy, convenience, reduced toxicity, reduced side effects and frequency of doses [2,3,4,5]. Key issues of preserving drug activity in producing protein/peptide formulation include the processes like cutting back on organic solvent use, lowering processing temperature and reducing initial burst release. As another critical element of CDDS, drug carriers, being excreted or biodegraded within a reasonable time course after the complete release of drugs with no risk of cellular or organ accumulation, would be appreciated. 

Hydrogels, as a class of hydrophilic polymer maintaining large amounts of water and exhibiting a semi-solid morphology, have been extensively studied and applied to the fields of wound treatment, cell therapies, tissue engineering [6,7] as well as drug carriers [8,9,10]. In particular, PEG-based thermo-sensitive hydrogels prepared from ABA-type triblock copolymers are considered as promising carriers of chemical and protein drugs for their rapid sol-gel transition [11,12,13,14]. This intriguing feature allows loading drugs into sol solutions at room temperature by simple mixing and then forming gel depots at body temperature. The avoidance of severe temperature condition and usage of organic solvents during preparation of formulation is beneficial to preserve the activity of sensitive drugs. Although plenty of promising results have been accomplished for thermo-responsive hydrogels [15,16], some shortcomings still remained unresolved to restrict their practical applications in drug delivery. A typical problem is the uncontrolled and fast release of hydrophilic and small molecular drugs with a disastrous initial burst. Moreover, the present available hydrogels usually exhibit low mechanical strength, which will weaken the structure stability when implanted into living body. 

Temperature dependent sol-gel transition behavior and mechanical properties of polymer hydrogels can be intensively influenced by their end groups [17,18,19,20]. B.B. Wang et al. reported an acrylate terminated PCL–PEO–PCL to give a chemical cross-linked hydrogel for peptide delivery. [21] Peptide end capped PCLA–PEG–PCLA thermo-sensitive hydrogel was reported by Xun et al. [22] to give the results that functionalized hydrogels displayed improved mechanical properties for hydrogen-bonds among amino groups. 

**Figure 1:**
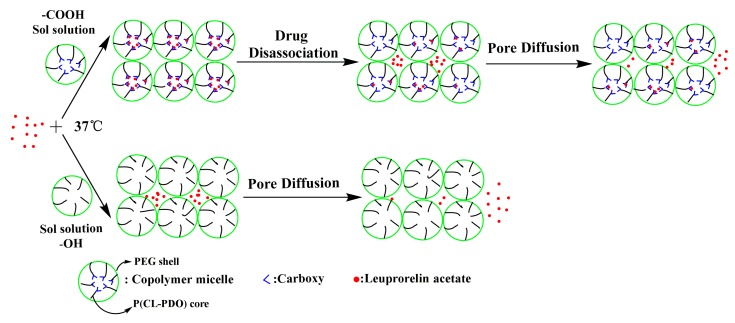
Comparison of release mechanisms for hydroxyl terminated (–OH) and carboxylic terminated (–COOH) PECP hydrogels loaded with leuprorelin acetate.

Herein, we have synthesized the biodegradable triblock copolymer poly (ε-caprolactone-co-p-dioxanone)-b-poly (ethylene glycol)-b-poly (ε-caprolactone-co-p-dioxanone) [P(CL–PDO)–PEG–P(CL–PDO)] and then terminated it with succinic anhydride for the first time. The positive effects of carboxylic end groups on both phase transition behaviors and thermodynamic properties are discussed. Moreover, the performance of peptide drug release in vitro is compared between PECP and its modified derivative; only simple mixing is needed to have drug loaded into the hydrogels at mild temperature condition. We were delighted to find that the ionic interaction between peptide drug and copolymer carboxyl end group has effectively extended the releasing period and restrained the initial burst effect (Figure 1).

## 2. Materials and Methods

### 2.1. Materials

PEG1500 and succinic anhydride (SA) was purchased from Chengdu Kelong Chemical Corp. (Chengdu, China). ε-Caprolactone (CL) was purchased from Acros (Geel, Belgium) and distilled over CaH_2_ under reduced pressure prior to polymerization. p-dioxanone (PDO) was prepared in our lab according to a published procedure [23]. Stannous octoate [Sn(Oct)_2_] was purchased from SIGMA (St.Louis, MO, USA) and used directly. 1,6-diphenyl-1,3,5-hexatriene (DPH) was purchased from Acros and used without any further purification. Leuprorelin acetate was purchased from Astatech Trading Corporation (Chengdu, China).

### 2.2. Synthesis of P(CL–PDO)–PEG–P(CL–PDO) Triblock Copolymer

The P(CL–PDO)–PEG–P(CL–PDO) (PECP) triblock copolymers was prepared according to our previously published paper [24]. Briefly, PEG (M_w_ 1500) with appropriate amounts of ε-caprolactone and p-dioxanone (according to the desired feed ratio) were introduced into a dry flask at room temperature under nitrogen protection. The flask was then degassed under vacuum and replaced with nitrogen several times to remove residual water and oxygen in reaction system. Next, stannous octoate was added to the reaction mixture and stirred at 150 °C for 12 h. The crude product was purified by dissolution in dichloromethane and precipitation from diethyl ether. The residual solvent was removed under vacuum at 80 °C overnight.

### 2.3. Synthesis of Carboxyl Group Terminated PECP

SA (0.2 g, 2 mmol) was introduced into the reaction flask after previously purified PECP copolymers (5 g) were dissolved in 15 mL anhydrous pyridine, and then, under nitrogen protection, mixtures were reacted at room temperature. Seventy-two hours later, the raw product was dissolved in THF(tetrahydrofuran) and precipitated in diethyl ether several times before removing residual solvents under vacuum at 80 °C overnight.

### 2.4. ^1^H NMR Study

NMR spectra was recorded in CDCl_3_ on a Bruker ARX-300 NMR spectrometer (Rheinstetten, Germany) to investigate compositions of the synthesized copolymers. The chemical shifts (ppm) were referenced relative to tetramethylsilane (TMS, 0.00 ppm) as the internal reference.

### 2.5. Gel Permeation Chromatography

Polymer average molecular weights and their distributions were measured on a Waters Associates Model ALC/GPC 244 HPLC system (Milford, MA, USA) with Ultrastyragel Linear columns. The eluting solvent was THF at a flow rate of 1.0 mL/min at 35 °C.

### 2.6. Sol–Gel Transition

The sol–gel transition was determined by the test tube inversion method [25,26]. The temperature was increased by 1 °C per step. Vials with a diameter of 1.1 cm containing given concentrations of the copolymer solutions (ranging from 12% to 30%) were immersed in a water bath at the designated temperature for 20 min. The gel state was determined by visual observation (i.e., the gel state was attained when no macroscopic phase flow occurred upon inverting the tube for 1 min).

### 2.7. Critical Micelle Concentration (CMC)

The CMC values were determined by the dye solubilization method [25]. A 1,6-diphenyl-1,3,5-hexatriene (DPH) solution in methanol (10 μL at 0.4 mM) was injected into aqueous polymer solutions (10 mL) at various concentrations between 0.0035 wt % and 0.0001 wt %. Each mixed solution was then equilibrated for 6 h in the dark before being measured. Absorption spectra of samples were recorded from 330 to 400 nm at 20 °C on a Shimadzu UV-120 luminescence spectrometer (Kyoto, Japan). The absorbance at 356 nm was plotted against the polymer concentration, and the crossing point of the two extrapolated straight lines was defined as the critical micelle concentration.

### 2.8. Differential Scanning Calorimetry (DSC)

DSC was performed on Netzsch DSC 204 F1 (Netzsch GmbH, Selb, Germany) in an aluminum pan under a nitrogen atmosphere. To study the non-isothermal crystallization of the samples, the temperature was raised up to 80 °C and kept constant for 5 min to eliminate the thermal history. Cooling curves were recorded when scans were run from 80 °C to −60 °C at the rate of 10 °C/min. Then, the temperature was held constant for another 5 min before heating curves were recorded with scans run at 10 °C/min from 60 °C back to 80 °C.

### 2.9. Dynamic Rheological Analysis

The phase transition behavior of each aqueous polymer solution was determined using dynamic rheometry (Bohlin Gemini 2000, Malvern, UK). The aqueous polymer solution was placed between parallel plates (diameter, 40 mm) whose interval was adjusted to 0.5 mm. Under a controlled stress of 4.0 dyn·cm^−2^, the data were collected at an angular frequency of 1.0 rad/s. The heating rate was set at 0.5 °C/min.

### 2.10. Release of Peptide Drug

Five grams of PECP and its carboxyl modified derivative were separately dissolved in acetone; afterwards, to HOOC–PECP–COOH solution was added moderate NaOH aqueous solution, transforming the carboxyl end group to carboxylic sodium. Both of the solutions were then concentrated in vacuum oven to remove residual acetone before 10 mg leuprorelin acetate was added in. After that, appropriate amounts of double distilled water were put in to give a mixture solution with the copolymer concentration equaling to 25 wt %. Next, the two prepared mixture solutions were centrifuged at the rate of 4000 rad/min, and then their supernatants (1 g) were separately transferred into two 10 mL plastic tubes following up with incubation under 37 °C for 10 min to attain a gel formation. Eventually, 10 mL PBS buffer (pH = 7.4) at 37 °C was slowly injected into the two gel samples along the wall of tubes. Release samples on different dates were withdrawn and kept in a refrigerator for following up UV-Vis spectroscopy analysis. Each experiment was performed in triplicate and the results were calculated by averaging the data with standard deviation. 

### 2.11. UV–Vis Spectroscopy

The UV-Vis spectroscopy was carried out on a SCINCO: S-3130 UV-vis spectrophotometer (Seoul, Korea). The UV absorbance at 280 nm was first plotted against the drug concentration ranging from 0–0.5 mg/mL in standard solutions to give a reference curve. Then, after recording the UV absorbance at 280 nm of each withdrawn sample, the drug concentrations on different releasing dates were calculated according to the reference curve. Finally, we obtained the curves between accumulative amounts of released drug and sampling dates.

## 3. Results and Discussion

### 3.1. Synthesis and Characterization of PECP Triblock Copolymers

**Scheme 1:**
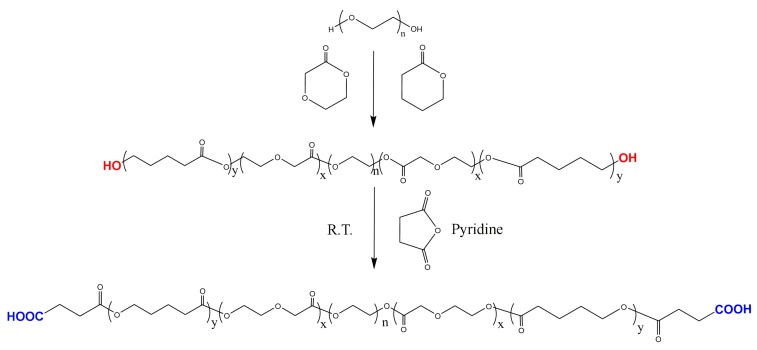
Synthetic scheme of hydroxyl terminated and carboxylic terminated PECP copolymers.

**Figure 2:**
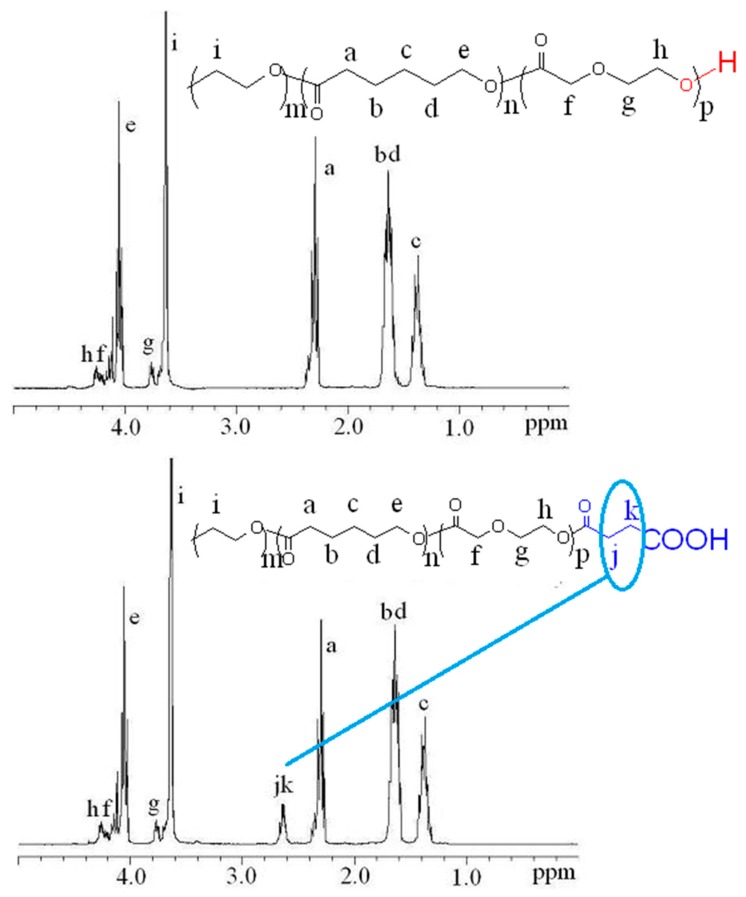
^1^H NMR spectra of PECP copolymers with hydroxyl (**Upper**) and carboxylic (**Lower**) end groups.

As shown in Scheme 1, the triblock copolymer PECP with hydroxyl end group was first synthesized by ring-opening polymerization of PDO and CL monomers in the presence of PEG with stannous octoate used as the catalyst. Carboxylic terminated HOOC–PECP–COOH copolymer was synthesized by coupling succinic anhydride to both chain ends of PECP. The two resulting copolymers were characterized by ^1^H NMR in Figure 2. For PECP copolymer (the upper one in Figure 2), the main characteristic peaks and corresponding structural units were summarized in supporting information (Table S1). Assigned to the methylene protons of PDO units, the characteristic peaks at around 4.33 ppm (2H, t) (h), 4.18 ppm (2H, s) (f), and 3.78 ppm (2H, t) (g) indicated the successful incorporation of PDO segments into the triblock copolymer. For HOOC–PECP–COOH (the lower one in Figure 2), a new group of peaks (p, k) at around 2.61–2.63 ppm came out to be the evidence that SA had been successfully coupled to PECP copolymer. By calculating integral areas of characteristic peaks, we arrived at a conclusion that more than 98% of PECP copolymers had been end capped by carboxyl groups.

### 3.2. Sol-Gel Transition Behaviors of PECP Copolymer Aqueous Solutions

**Figure 3:**
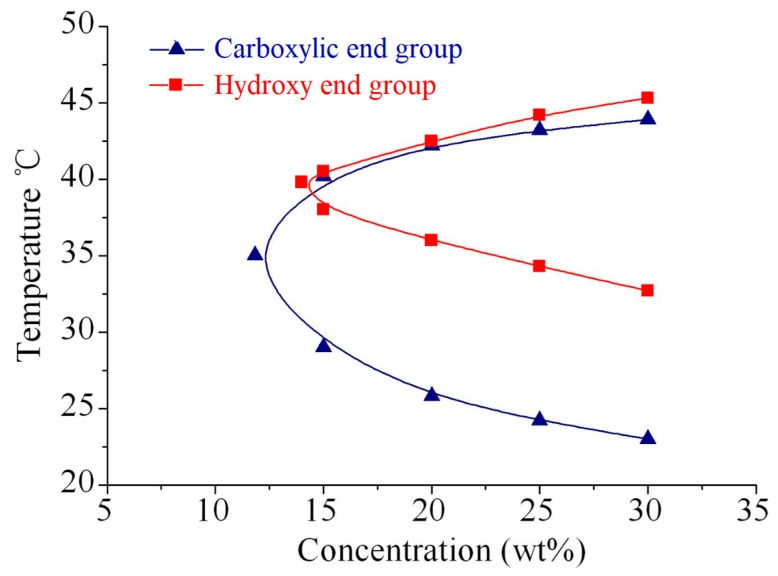
Phase transition curves of hydroxyl terminated and carboxylic terminated PECP copolymers.

Phase transition behavior of thermo-sensitive polymer aqueous solution could be influenced by multiple factors like ratio between hydrophilic and hydrophobic segments, PEG molecular weight and chemical microstructure. For PECP, its sol-gel transition behavior relating to the above structural factors was elaborately discussed in our previous work [19]. According to similar studies on PEG based amphiphilic block polyesters containing PCL component [27,28], such gelation behavior was mainly caused by the dehydration of PEG segments that generally resulted in the increased micelle size with the rising temperature. Here in this paper, we would like to see the influence of end groups on gelation behavior. As revealed in Figure 3, the sol-gel transition temperature of original PECP was decreased by nearly 10 °C after the terminal hydroxyl end group was converted to carboxyl. This phenomenon could be attributed to the enhanced interaction between polymer chains brought by carboxylic end groups at comparatively high polymer concentrations (12–35 wt %). From this point of view, tendency for those hydrophobic segments between different micelles to tangle together was increased. Therefore, when PEG was dehydrated with rising temperature, the increased inter-micelle interaction, brought by the presence of hydrogen bond of terminal COOH groups, led to the occurrence of gel formation under lower temperature. Meanwhile, it was expected that the end group modification would result in a larger size of aggregated micelles and more stable gel structure. 

**Figure 4:**
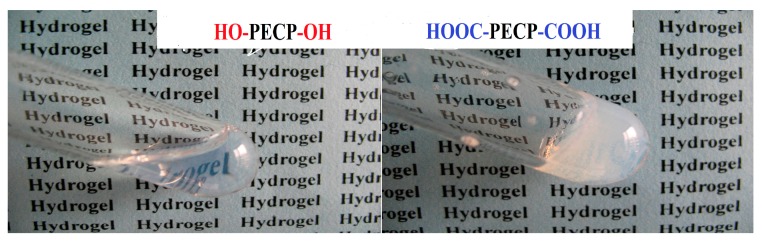
Hydroxyl terminated PECP sol solution (25 wt %) and carboxylic terminated PECP gel state (25 wt %) visualized at 30 °C.

Different phase states of PECP and HOOC–PECP–COOH aqueous solutions at the concentration of 25 wt % were presented in Figure 4. It was shown that at 30 °C, the HOOC–PECP–COOH polymer solution already turned into gel while the unmodified PECP aqueous solution was still in the sol state. Since both of the polymer aqueous systems could form into a stable hydrogel near 37 °C, it was possible to compare their drug release properties at body temperature in vitro.

### 3.3. Self-Assembly of Micelles

**Figure 5:**
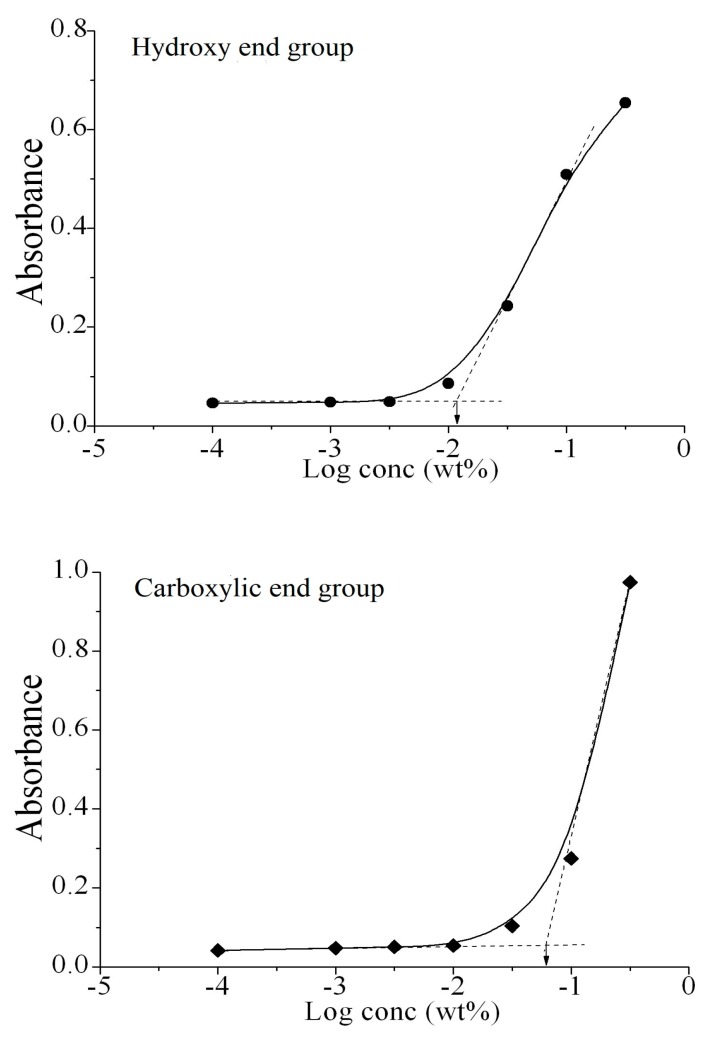
Critical micelle concentrations of hydroxyl terminated and carboxylic terminated PECP.

It was widely regarded that when heated to a higher temperature, the thermos-responsive copolymer molecules would self-assemble into a kind of hydrophobic association intermediate (micelle), which would then aggregate into hydrogel. To compare the tendency to form micelles, the CMC values for both copolymers were measured according to DPH method. It can be seen in Figure 5 that the CMC of carboxylic terminated PECP was a bit higher than that of hydroxyl terminated PECP. When the terminal hydroxyl of PECP was modified with succinic anhydride, the hydrophilicity of the copolymer was enhanced by the introduction of carboxylic groups. The increased hydrophilicity prevented the polymer molecules from aggregating into micelles, leading to a higher CMC value. The CMC results seemed not to be in agreement with the sol-gel transition temperatures presented in Figure 3. A possible explanation for this contradiction lies in the different levels of assembly behavior of copolymer molecules. The CMC value was related to the assembly event that occurred from single molecules to micelles at the nanoscale level, while the sol-gel transition temperature was in connection with the aggregation happening during the transformation from micelles to macroscopic status. For the former case, under very diluted solution, the hydrophilicity of COOH terminal group played an obvious role for single molecules, which led to a decreased CMC value. While for the latter case at high copolymer concentration, the hydrogen bond between different copolymer chains became predominant, resulting in the obvious decrease of sol-gel transition temperature. Similar contradiction was also reported in other thermogelling system in which the copolymer chain end was modified by a peptide [22]. Therefore, if the terminal COOH group is replaced by other hydrogen bond group, such as NH_2_, or other similar hydrophilic comonomer is introduced in the hydrophobic block, we expect that similar CMC and sol-gel transition results could be repeated as well. 

### 3.4. Crystallization Behaviors of PECP Copolymers

**Figure 6:**
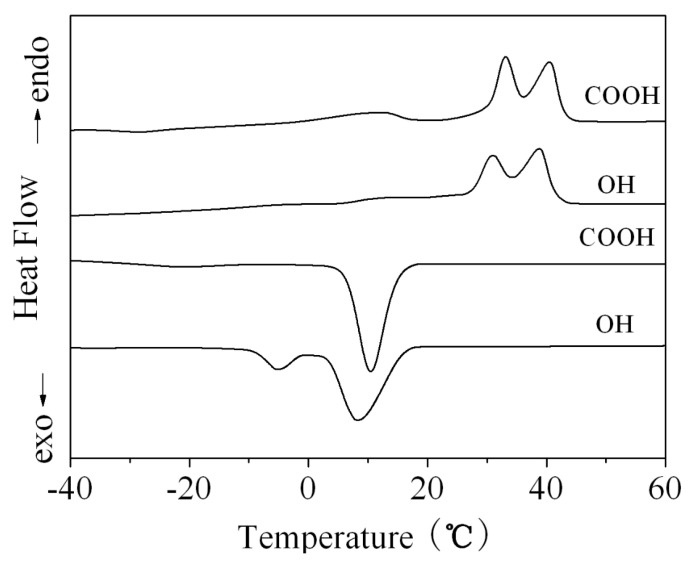
DSC thermograms of hydroxyl terminated and carboxylic terminated PECP copolymers.

The DSC thermograms for the two compared copolymers were presented in Figure 6. The crystallization peaks located between 0 °C and 20 °C in cooling curves were assigned to the PCL hydrophobic segments. It was found that the crystallization point of carboxylic terminated PECP was ahead of that point of unmodified PECP with a much narrower and sharper peak. This result indicated that the carboxylic termination had enhanced the crystallization capability of PECP hydrophobic chains. The as-formed and well-organized crystallized domains allowed little opportunity for amorphous PEG chains to recrystallize during the cooling curve, therefore, the crystallization peak between −10 °C and 0 °C attributed to the PEG chains of PECP disappeared after carboxylic termination. From the heating curves, we found that the melting point had been delayed by carboxylic termination, which agreed with the enhanced crystallization behavior. The exhibited enhanced crystallization capability of carboxylic terminated PECP was potentially useful to improve the solidification of the formed hydrogel.

### 3.5. Dynamic Mechanic Analysis

**Figure 7:**
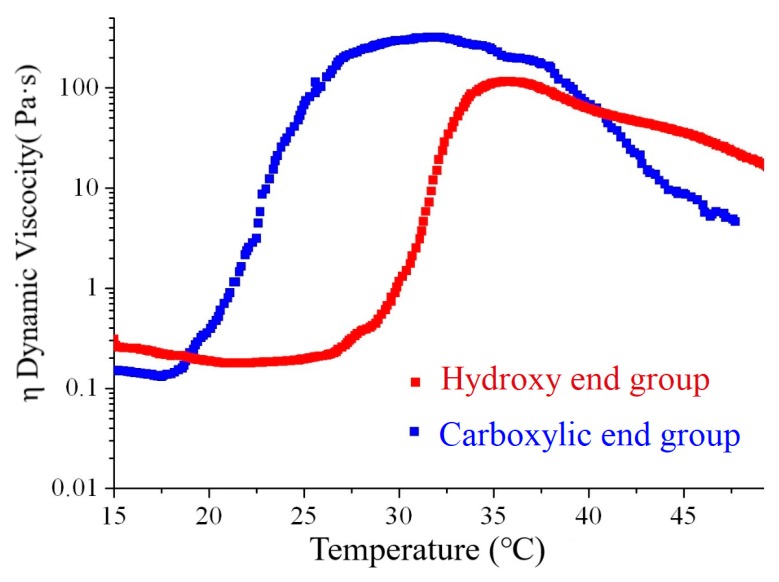
Dynamic mechanical analysis of hydroxyl terminated and carboxylic terminated PECP copolymer solutions (25 wt %) as a function of temperature.

The dynamic viscosity changes of polymer solutions with temperature at the concentration of 25 wt % were exhibited in Figure 7. Both solutions had shown very low viscosities in the sol state at low temperature, which was beneficial for injectable use through thin needles. When heating the solution, viscosity of carboxylic terminated sample quickly reached its top value over 300 Pa·s near 30 °C. By contrast, the viscosity of unmodified PECP aqueous solution had a viscosity below 100 Pa·s at the gel point of 35 °C. The enhanced viscosity of the gel could be ascribed to the strong intermolecular hydrogen bond force between polymer chains after carboxylic termination. So, it was convinced to say that end group modification had improved the solidification of temperature-dependent hydrogels formed by physical crosslinking. In our previous work, similar P(CL–GA)–PEG–P(CL–GA) hydrogel with a lower viscosity of 14.9 Pa·s was stable for practical use and the physical integrity could preserve for at least four weeks at 37 °C in PBS buffer under the shaken condition of 60 rpm [28]. Therefore, both OH–PECP–OH and HOOC–PECP–COOH hydrogels with higher viscosities at the gel temperature are expected to be mechanically stable. Actually, in the latter release experiments, we found that both PECP hydrogels were physically stable for at least one month. 

### 3.6. Drug Release Behaviours In Vitro

Leuprorelin acetate was clinically applied to treating diseases like prostatic carcinoma, endometriosis and uterine fibroid. However, the problem of low permeability through biofilms and vulnerability in the alimentary tract, being decomposed by enzymes for instance, brought about low drug bioavailability through traditional oral, nasal and rectal administration routes. For this reason and to reduce the dose frequency, it was necessary to develop sustained drug release formulation that could be directly implanted into living body. Though PLGA sustained microspheres are already available for clinical application, the production processes were complex and not beneficial for preserving the activity of protein drugs [29]. 

**Figure 8:**
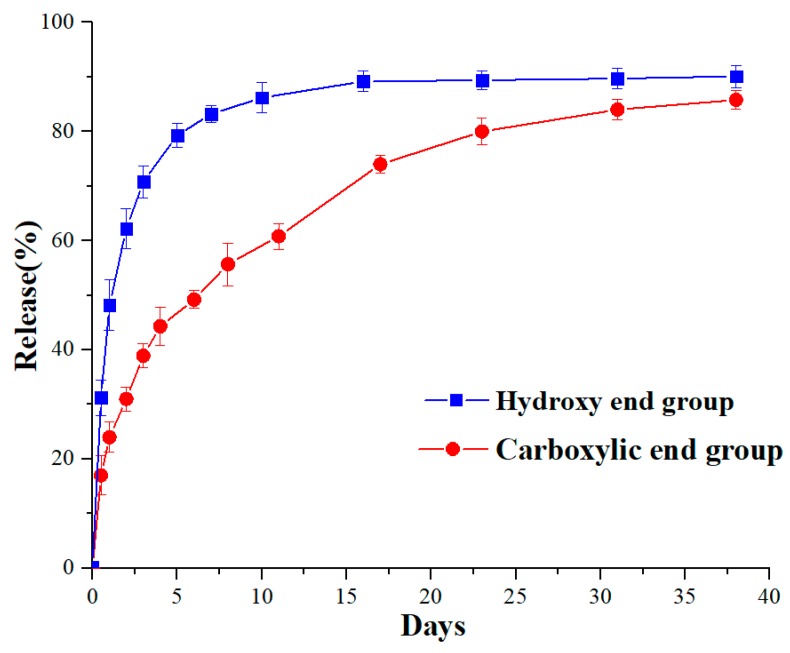
In vitro release profiles of Leuprorelin acetate from hydroxyl terminated PECP hydrogel and carboxylic terminated PECP hydrogel.

Hydrogels of both PECP and its derivative containing 0.2 wt % of leuprorelin acetate were prepared, and their accumulative amounts of released drug measured in PBS buffer were plotted against sampling date (Figure 8). The release media was neutral, which is similar to that of body injection site since the applied gel formulation was generally administered by intramuscular or subcutaneous injection. It was shown that drug releasing rate had been significantly slowed down after carboxylic termination with 50% of loaded drug remaining unreleased after one week, and the whole releasing period was even extended to more than one month. Since all the PECP components including PEG, CL and PDO used to synthesize the copolymer have already been approved by FDA for clinical use, we expect that the hydrogel has good biocompatibility.

The improvement in release profile was believed to be the function of retarded mechanism of drug release from the derivative HOOC–PECP–COOH hydrogel (Figure 1). Similar to other thermo-responsive hydrogels, small molecular and hydrophilic leuprorelin acetate mainly located within the loose and hydrophilic PEG pores of the original PECP hydrogel, leading to free and fast diffusion mechanism. However, for the modified HOOC–PECP–COOH hydrogel, leuprorelin acetate could be coupled to carboxyl terminated PECP by exchanging acetate at neutralized condition. The formed polymer-drug conjugate pair, as a driving force, allowed enhanced solubility of drug molecules into the hydrophobic P(CL–PDO) micro-domains. On the drug releasing stage, the resistance to disrupt the polymer-drug ionic interaction and the slow speed of drug molecules to penetrate out from the compact and hydrophobic micelle cores accounted for the retarded release mechanism. This strong ionic interaction was effective at suppressing the initial burst, which made carboxyl terminated PECP a promising drug delivery material.

## 4. Conclusions

A novel thermo-responsive PECP triblock copolymer with carboxylic acid end groups was prepared for the first time by end capping with succinic anhydride. This carboxylic termination had enhanced intermolecular force among polymer chains by forming hydrogen bond. As a result of this, the sol-gel transition temperature was remarkably decreased along with the improvement of hydrogel mechanical strength. Furthermore, the peptide drug, leuprorelin acetate, could form conjugate pair with polymer chains via strong ionic interaction, which increased the solubility of the drug into the compact and hydrophobic domains of the HOOC–PECP–COOH hydrogel. This led to the retarded and sustained release of drug over one month with weak burst release effect. So, this new carboxylic terminated PECP hydrogel was a promising delivery carrier to develop injectable formulations for peptide drugs that had sustained release profile. 

## Supplementary Materials

The following are available online at http://www.mdpi.com/1996-1944/11/3/338/s1, Table S1. Characteristic 1H NMR peaks for PECP copolymers.
