# Supplementary material for: Carboxylic Terminated Thermo-Responsive Copolymer Hydrogel and Improvement in Peptide Release Profile
Source: Materials (Basel). 2018 Feb 26;11(3):338. doi: 10.3390/ma11030338 (PMC5872917; doi:10.3390/ma11030338)
Supplement: Supplementary file 1 [file materials-11-00338-s001.pdf]

Supporting Information

Table S1 Characteristic ^{1}H NMR peaks for PECP copolymers.

Peak no.	Structure unit	Peak position (ppm)	Label in Figure 2
**1**	-O**CH$_2$**CH$_2$O-	3.60-3.64	i
**2**	-COCH$_2$CH$_2$CH$_2$CH$_2$**CH$_2$**O-	4.02-4.07	e
**3**	-COCH$_2$CH$_2$**CH$_2$**CH$_2$CH$_2$O-	1.30-1.42	c
**4**	-COCH$_2$**CH$_2$**CH$_2$**CH$_2$**CH$_2$O-	1.56-1.68	b, d
**5**	-CO**CH$_2$**CH$_2$CH$_2$CH$_2$CH$_2$O-	2.25-2.32	a
**6**	-COCH$_2$OCH$_2$**CH$_2$**O-	4.30-4.35	h
**7**	-COCH$_2$O**CH$_2$**CH$_2$O-	3.70-3.82	g
**8**	-CO**CH$_2$**OCH$_2$CH$_2$O-	4.17-4.22	f
